# Healthcare Services Utilisation and Financial Burden among Vietnamese Older People and Their Households

**DOI:** 10.3390/ijerph20126097

**Published:** 2023-06-10

**Authors:** Long Thanh Giang, Tham Hong Thi Pham, Phong Manh Phi, Nam Truong Nguyen

**Affiliations:** 1 Faculty of Economics, National Economics University (NEU), Hanoi 11616, Vietnam; 2 Faculty of Mathematical Economics, National Economics University (NEU), Hanoi 11616, Vietnam; 3 Faculty of Political Studies, Hanoi University of Mining and Geology (HUMG), Hanoi 10000, Vietnam; 4 Institute of Social and Medical Studies (ISMS), Hanoi 10000, Vietnam

**Keywords:** healthcare, older people, out-of-pocket payments, social health insurance, Vietnam

## Abstract

Background: This research examined differences in the utilisation of healthcare services and financial burden between and within insured and uninsured older persons and their households under the social health insurance scheme in Vietnam. Methods: We used nationally representative data from the Vietnam Household Living Standard Survey (VHLSS) conducted in 2014. We applied the World Health Organization (WHO)’s financial indicators in healthcare to provide cross-tabulations and comparisons for insured and uninsured older persons along with their individual and household characteristics (such as age groups, gender, ethnicity, per-capita household expenditure quintiles, and place of residence). Results: We found that social health insurance was beneficial to the insured in comparison with the uninsured in terms of utilization of healthcare services and financial burden. However, between and within these two groups, more vulnerable groups (i.e., ethnic minorities and rural persons) had lower utilization rates and higher rates of catastrophic spending than the better groups (i.e., Kinh and urban persons). Conclusion: Given the rapidly ageing population under low middle-income status and the “double burden of diseases”, this paper suggested that Vietnam reform the healthcare system and social health insurance so as to provide more equitable utilisation and financial protection to all older persons, including improving the quality of healthcare at the grassroots level and reducing the burden on the provincial/central health level; improving human resources for the grassroots healthcare facilities; encroaching public–private partnerships (PPPs) in the healthcare service provision; and developing a nationwide family doctor network.

## 1. Introduction

The population of Vietnam entered the “ageing” phase in 2011 when the older population accounted for 10 per cent of the total population [1]. Given its low middle-income position as defined by the World Bank, Vietnam is experiencing a much faster-ageing population than other countries. For example, it is expected to take Vietnam less than 20 years to reach the “aged” phase, which is much shorter than many industrialised countries such as France (115 years), Sweden (50 years), and Japan (26 years). This time will be even faster than Thailand, which has been considered a fast-ageing population [1,2,3,4].

Along with this demographic transition, Vietnam has faced various challenges in healthcare for older persons who face the “double burden of diseases”, in which non-communicable and chronic diseases (such as diabetes, cardiovascular disease, and osteoarthritis) have become more popular [1,2,5,6,7,8]. These diseases require long-term and costly treatments for older persons. Many older persons are prevented from accessing and utilising healthcare services due to a large financial burden for medical treatments, as well as transportation, accommodation, and other related costs for inpatient care [8,9]. Such a situation may result in catastrophic health expenditures (*CHEs*), which is when out-of-pocket payments account for more than 40 per cent of a household’s total income without subsistence spending, and *CHEs* in turn may push older persons and their families into impoverishment. In such a context, social health insurance is an important financial tool to help people in general and older persons, in particular, to access healthcare services more easily since it helps reduce the related financial burden [10,11].

Social health insurance (SHI) in Vietnam has been a tool to develop a sustainable, equitable, and effective health financing system so as to achieve universal health coverage (UHC) by 2030 under the slogan “No one must be left behind” [11,12]. In the past three decades, the Government of Vietnam (GOV) has conducted various health reforms in order to expand health insurance coverage so as to strengthen the financial protection of the healthcare system. The SHI was first introduced in 1992 under Decree 299/HDBT, dated 15 August 1992, with the implementation of a compulsory scheme for civil servants and pensioners. Under Decision 139/2002/QD-TTg, dated 15 October 2002, the Healthcare Fund for the Poor was introduced, where the poor and ethnic minorities had SHI premium exemption and medical treatments. Decree 63/2005/ND-CP, dated 16 May 2005, expanded the compulsory groups, particularly, children aged under six had free medical treatments, and the poor and ethnic minorities were fully subsidised by the government budget in purchasing SHI cards. The SHI Law was first introduced in 2008 under Law No. 25/2008/QH12, dated 14 November 2008, and it was amended in 2014 under Law No. 46/2014/QH13, dated 13 June 2014, with various revisions to the contribution rate, level of subsidies, household-based participation, levels of co-payment, provider payment methods, and benefits packages [11,12]. The next amended SHI Law is expected to be approved in 2023, in which designing a sustainable financing mechanism via SHI is being discussed; thus, it is necessary to review the situation of SHI in the first amended Law, especially the issues related to *CHEs* and impoverishment in utilising healthcare services.

A number of studies have been conducted to estimate the incidence of *CHEs* and impoverishment in Vietnam (see, for instance, [12,13,14,15,16]). Using data sets from the Vietnam Household Living Standard Survey (VHLSS) conducted in 1993 and 1998, [13] found that *CHEs* in Vietnam decreased from 9.3 per cent to 7.8 per cent in this period. Using VHLSS data sets from 2002 to 2010, [14] showed that the *CHE* rate varied from around 4 to 6 per cent and the impoverishment rate ranged from about 3 to 4 per cent. [9], using annual data from the SHI-paid healthcare services provided by the Vietnam Social Security Agency (VSS) in 2012–2016, found that SHI could reduce healthcare costs for older persons, particularly for inpatient admissions. Using the same data source, [15] showed that SHI brought benefits to many people by increasing their accessibility to healthcare services and reducing the burden of paying for these services, especially those with chronic diseases. [16], using data collected from Cao Lanh district, Dong Thap province (Vietnam), for 1143 individuals using outpatient care, showed that SHI reduced average out-of-pocket payments (*OOPs*) by about 21 per cent and that using private health facilities incurred more *OOPs* than public health facilities.

None of these studies, however, discussed these important issues for older persons and their households. As such, this study was expected to contribute to the existing literature by using the VHLSS conducted in 2014—the year that the first amended SHI Law was introduced—to explore and compare the situation of *CHEs* and impoverishment in utilising healthcare services between insured and uninsured older persons along with their individual and household characteristics. The findings of this research provide important policy implications for the GOV and its line ministries in health-related sectors when revising the SHI Law so as to provide accessible, affordable, and adequate healthcare services to older persons—an increasing population in Vietnam—now and in the coming decades.

## 2. Materials and Methods

### 2.1. Data

This research used data from the VHLSS conducted in 2014 to explore the situation for older persons—both the insured and the uninsured. The VHLSS has been conducted by GSO since 1992. The VHLSS 2014 was conducted nationwide with a sample size of 46,995 households in 3133 communes/wards, which were representative at national, regional, urban–rural, and provincial levels. A total of 37,596 households were asked about income and other issues (module 1), and 9399 households were asked about income, expenditure, and other issues (module 2) [17]. To calculate *CHEs* and other indicators, this study used module 2. Because the age of each individual in each surveyed household was known, it was possible to identify who were older persons (those aged 60 years and over, as defined in the Law on the Elderly of Vietnam in 2009) as well as their households (those having at least one older person). In module 2, the number of households with at least 1 older person (namely, elderly households) was 3601, and the number of older persons was 4165.

The survey collected information at the household level (such as income, expenditure, and housing status), as well as at the individual level (such as age, gender, the highest education level, marital status, and employment status).

In regard to the utilisation of healthcare services and participation and the usage of SHI, the VHLSS collected information about the health status of the interviewees and their use of healthcare services (such as the number of inpatient admissions and outpatient visits and their respective costs). Therefore, it was possible for us to define who were the insured (who had an SHI card) and the uninsured (who did not have any SHI card) older persons and how they used healthcare services and the respective costs.

### 2.2. Methods

To pursue the research objectives, we disaggregated the older population into different socio-economic characteristics such as age, gender, ethnicity, and place of residence (urban vs. rural areas). In all estimates, we used the sample weights for respective household- or individual-level data so as to make the results representative of the older population. Stata version 14 was used for all calculations.

We used definitions from the World Health Organization (WHO) [18] on catastrophic health expenditures (*CHEs*) and impoverishment. *CHEs* are *OOPs* that exceed a predefined percentage or threshold of a household’s resources, which can push households into poverty and impoverishment. The predefined percentage could be 10 per cent to 40 per cent, depending on the research purposes [14,18,19].

Financial burdens in healthcare are measured at the household level and calculated using *OOPs* as a percentage of a household’s capacity to pay. *OOPs* are calculated using all spending for the healthcare of a household in the past 12 months. Typically, *OOPs* include doctor’s consultation fees, medication purchases, and hospital bills. Although spending on alternative and/or traditional medicine is included in *OOPs*, expenditure on health-related transportation and special nutrition are excluded. *OOPs* are net of SHI reimbursement.

The capacity to pay (*ctp*) of a household is measured using total income without subsistence spending. A household’s subsistence spending is the minimum requirement to maintain basic life in a society. A poverty line is used in this study as subsistence spending. In particular, the poverty line is defined as the food expenditure of households whose food expenditure share of the total household expenditure is at the 50th percentile in the country. In order to minimize measurement error, we used the average food expenditures of households whose food expenditures share of the total household expenditures was within the 45th and 55th percentile of the total sample. Considering the economic scale of household consumption, the household equivalence scale was used rather than the actual household size. We applied a scale of 0.56 as suggested by the WHO for a study based on household survey data from 59 countries [19]. Since some households might declare that food spending was lower than subsistence income, the capacity to pay (*ctp*) for a household with at least an older person can be measured as follows:

*ctp_h_* = *exp_h_* − *se_h_* if *se_h_* < *f_h_* and *ctp_h_* = *exp_h_* − *f_h_* if *se_h_* > *f_h_*, (1)

where, for a household *h*, *ctp_h_* is the household’s capacity to pay; *exp_h_* is the total household spending; *se_h_* is the household’s subsistence spending; and *f_h_* is the household’s food spending.

Thus, the financial burden of healthcare for the household *h* with at least one older person (*fb_h_*) is measured as follows:

(2) $$fb_{h}=\frac{OOP_{h}}{ctp_{h}}\cdot 100\left(\%\right)$$

Along with financial burden, an elderly household could be at risk of impoverishment due to high payments for healthcare services. A non-poor household is impoverished by health payments when it becomes poor after paying for health services based on the poverty line. The probability that an elderly household *h* will become impoverished can be estimated as follows:

*impov* = 1 if *exp_h_* > *se_h_* and *exp_h_* − *OOP_h_* < *se_h_*; and otherwise, (3)

where *impov* represents the probability that the household *h* with at least one older person will become impoverished after paying for healthcare services.

### 2.3. Hypotheses

Following the main objective to compare the utilisation of healthcare services between insured and uninsured older persons and their households in terms of *CHEs* and their relevant indicators, this research will test the following hypotheses.

**Hypothesis** **1.** *For both inpatient and outpatient healthcare services, insured older persons have higher utilisation rates than their uninsured counterparts, particularly when considering their specific characteristics such as age, gender, residential place, and ethnicity.*

**Hypothesis** **2.** *Uninsured older persons generally have higher OOPs than their insured counterparts.*

**Hypothesis** **3.** *In general, for both insured and uninsured older persons, the more vulnerable groups (ethnic minorities, rural, and the poor) bear higher rates of OOPs than their counterparts.*

## 3. Results

Table 1 analyses and compares the frequency of using outpatient and inpatient healthcare services by older persons with (the insured) and without health insurance (the uninsured).

For outpatient healthcare services, the insured had a 25.86 per cent higher frequency of using services than the uninsured. In terms of age groups, the insured young-old (those aged 60–69) had a frequency of using services at 38.37 per cent higher than their uninsured counterparts (3.57 visits vs. 2.58 visits, respectively), and the difference was only 2.29 per cent among the oldest-old (those aged 80 years and above) (3.57 visits vs. 3.49 visits, respectively).

Regarding the ethnicity of the household head, Table 1 shows that, among households headed by the ethnic majority, i.e., Kinh persons, the insured older person had a service use rate that was 7.23 per cent lower than their uninsured counterparts (3.21 visits vs. 3.46 visits, respectively). The situation was contrasted among older persons living in households headed by Kinh (ethnic majority) persons: the insured had a 53.31 per cent higher frequency of using services than their uninsured counterparts (non-Kinh older persons) (4.4 visits vs. 2.87 visits).

In both urban and rural areas, insured persons had lower rates of using services than uninsured persons. In particular, the urban insured had a 26.07 per cent lower frequency of using services than the uninsured (3.49 visits vs. 4.40 visits, respectively), while the rural insured had a 22.05 per cent lower frequency of using services than the uninsured (2.63 visits vs. 3.21 visits, respectively).

In terms of the per capita expenditure quintile, the richest insured had a 36.80 per cent higher frequency of using services than the richest uninsured (4.61 visits vs. 3.37 visits, respectively). Among the poorest and the near-poor, the insured also had 22.55 per cent and 10 per cent higher service usage rates, respectively, than the uninsured.

With regard to the health facilities, Table 1 implies that the insured had a much higher rate of having services at central-level health facilities—those providing the highest quality of services—than their uninsured counterparts: the difference between these two groups was about 190 per cent. The difference between the insured and the uninsured was 47.89 per cent in district-level facilities, 29.34 per cent in provincial-level facilities, and 28.30 per cent in commune-level facilities. In contrast, for private facilities and other types of facilities, the insured had lower rates of using services than the uninsured (by 10.34 per cent and 16.17 per cent, respectively).

For inpatient healthcare services, Table 1 shows that insured persons generally had more than twice a higher frequency of using services than their uninsured counterparts (0.93 admissions vs. 0.27 admissions, respectively). In terms of other socio-economic characteristics, Table 1 indicates that the insured had a significantly higher frequency of service use than the uninsured. Particularly, for the older ages, there were higher differences in inpatient admissions between the insured and the uninsured. For the oldest-old, the gap was three times (1.16 admissions vs. 0.29 admissions, respectively), while those for the middle old and the young old were 1.8 times (0.83 admissions vs. 0.3 admissions) and about 2.5 times (0.88 admissions vs. 0.25 admissions), respectively.

Among Kinh persons, the insured had a 2.6 times higher frequency of using inpatient services than the uninsured, while the gap was about 1.4 times among ethnic minority persons. The same trends were observed in regard to place of residence: the gaps in using inpatient services between the insured and the uninsured in urban areas and rural areas were 3.3 times and 0.2 times, respectively.

In terms of the per capita expenditure quintile, the gap in utilizing inpatient services between the insured and the uninsured among the richest (0.84 times) was much smaller than those for the poorest, the near-poor, the middle, and the near-rich groups (3.41 times, 3.61 times, 2.52 times, and 3.33 times, respectively).

Significant differences were observed in all types of health facilities, in which the insured had significantly higher rates of using inpatient services in different health facilities than their uninsured counterparts.

Table 2 presents the results on the average out-of-pocket payments (*OOPs*) per one outpatient visit and one inpatient admission for insured and uninsured older persons.

For outpatient visits, the insured had an average OOP that was 30.43 per cent lower than that of the uninsured (VND 860,980 (Vietnam dong) vs. VND 1237,610, respectively).

In terms of age group, the *OOP* gaps between the insured and the uninsured were more significant as their ages increased: among the oldest-old, the insured had a lower average OOP of 63.58 per cent than the uninsured. The numbers were 24.45 per cent and 14.74 per cent for the young-old and the middle-old, respectively.

Among ethnic minority persons, the insured had less financial burden than their uninsured counterparts, as they had an average *OOP* that was 60.39 per cent lower. The difference among Kinh persons was 27.50 per cent.

The insured in both urban and rural areas had lower average *OOPs* than the uninsured, at 33.48 per cent and 31.23 per cent, respectively.

Similarly, at all per capita expenditure quintiles, the insured had lower average *OOPs* than the uninsured, and it was quite high among the poorest (48.61 per cent).

In terms of health facilities, the uninsured persons had much higher average *OOPs* than the insured, and the differences increased along with higher technical levels of health facilities. Notably, the gaps between provincial and central hospitals were very high, at around 45 per cent. In contrast, the insured had higher average *OOPs* when using services at private hospitals than the uninsured, partly because SHI has not been popularly applied to private facilities.

For inpatient admissions, the insured generally had much lower average *OOPs* than the uninsured (about 41.22 per cent or VND 3877 thousand vs. VND 6596 thousand, respectively). Among the middle-old, the insured had a 72.42 per cent lower average OOP than the uninsured, and this figure was only 26.29 per cent among the young-old. It was surprising to observe that the uninsured oldest-old had a 43.59 per cent lower average *OOP* than their insured counterparts, and this could be partly explained by the fact that the rate of using inpatient services of the former was much lower than that of the latter (as presented in Table 1).

For both Kinh and ethnic minority groups, the insured had lower average *OOPs* than the uninsured. However, the difference among the ethnic minorities was much lower than that of Kinh persons (10.68 per cent vs. 41.43 per cent, respectively).

Similarly, the insured in both urban and rural areas had lower average *OOPs* than their respective uninsured counterparts, but the difference was much higher in urban areas than in rural areas (57.47 per cent vs. 34.42 per cent, respectively). This could be explained by the fact that those living in urban areas usually use inpatient services at provincial and central hospitals, which are usually more costly than those in rural areas with mostly lower technical-level health facilities (i.e., district hospitals).

In regard to the per capita expenditure quintiles, except for the poorest, the insured generally had lower average *OOPs* than the uninsured, and the difference was higher for the richer. Among the poorest, the situation could be elucidated by the fact that the uninsured had a much lower rate of using inpatient services than the insured (as presented in Table 1).

Regarding health facilities, the insured had 22.70 per cent and 22.12 per cent lower average *OOPs* than the uninsured when they used inpatient services at district and provincial levels, respectively. This number increased sharply at the central level (50.98 per cent). Meanwhile, the insured had about 2.2 times and 2.8 times higher average *OOPs* than the uninsured when using inpatient services at the commune level and other health facilities, respectively. This could be explained by huge differences in the rates of using services in these facilities, as indicated in Table 1.

Table 3 describes the per capita *OOP*, the ratio of *OOP* to the ability to pay (*OOP*/*CTP*), and the ratio of *OOP* to the total expenditure of elderly households (*OOP*/*EX*). The results show that all these indicators for the insured elderly households were higher than those for their uninsured counterparts (VND 2937.88 thousand vs. VND 2200.82 thousand for the per capita *OOP*; 6.58 per cent vs. 5.08 per cent for the *OOP*/*CPT* ratio; and 4.07 per cent vs. 3.08 per cent for the *OOP*/*EX* ratio).

The results for the insured and the uninsured in regard to different characteristics (ethnicity of household head, place of residence, and expenditure quintile) show that the insured had higher per capita *OOP*, *OOP*/*CTP*, and *OOP*/*EX* than the uninsured. However, it is also noteworthy that both the insured and uninsured had a higher per capita *OOP* in urban areas than in rural areas. These differences could be explained by differences in household spending and utilization of both outpatient and inpatient services in the two areas by the insured and uninsured, as presented in Table 1 and Table 2.

Table 4 presents different thresholds of the *OOP*/*CTP* ratio in households with insured and uninsured older persons. There are four thresholds, i.e., more than 10 per cent; more than 20 per cent; more than 30 per cent; and more than 40 per cent, which are usually used in the WHO’s reports. The last range (more than 40 per cent) is considered as the one when an elderly household faces a catastrophic healthcare expenditure (*CHE*).

The results generally show that, at all thresholds, households with insured older persons had higher *OOP*/*CTP* ratios than those with uninsured older persons. More specifically, 2.42 per cent of households with insured older persons faced a *CHE*, while this rate was 1.29 per cent for households with uninsured older persons. For the households headed by Kinh and other ethnicities, there was a slight difference in the rate of households with a *CHE* among the insured older persons (2.44 per cent vs. 2.24 per cent), but the difference was large among the uninsured older persons (0.01 per cent vs. 1.34 per cent).

It is critical to see that the rate of households facing a *CHE*—either for the insured or uninsured older persons—in rural areas was significantly higher than in urban areas (2.95 per cent vs. 1.40 per cent for the insured; and 1.50 per cent vs. 0.78 per cent for the uninsured).

In terms of the per capita expenditure quintile, at the first threshold (i.e., more than 10 per cent), the rates of households with the uninsured were significantly higher than those of households with the insured. For catastrophic spending (i.e., *OOP*/*CTP* of more than 40 per cent), the poorest households with insured older persons and the middle households with uninsured older persons had the highest rates (3.04 per cent and 2.17 per cent, respectively).

Table 5 presents the percentage of households with insured and uninsured older persons that became impoverished due to healthcare spending (i.e., when the spending for healthcare was more than 40 per cent of the household’s capacity to pay). There were 1.77 per cent of such households for the insured, while there were 1.04 per cent of such households for the uninsured. Critically, for both insured and uninsured older persons, households headed by ethnic minority persons and those located in rural areas had significantly higher rates of becoming impoverished than those headed by Kinh persons and those located in urban areas, respectively.

## 4. Discussion

The above findings have demonstrated that SHI has been generally beneficial for older persons in Vietnam. In particular, the insured had a higher frequency of using outpatient and inpatient services at 25.86 per cent and 244.44 per cent, respectively, than the uninsured. This result was quite similar to those for the general Vietnamese population as well as for other countries. For example, [20] showed that SHI for the poor in Vietnam increased the likelihood of using outpatient services by 16 per cent and inpatient services by 30 per cent. [21] found that voluntary social health insurance in Vietnam helped the insured to increase the annual rate of outpatient visits and inpatient admissions by about 43 per cent and 63 per cent, respectively. Research on Taiwanese older persons by [22] indicated that the National Health Insurance Program (NHI) helped older persons increase their use of outpatient services by 14.18 per cent and their use of inpatient services by 9.05 per cent. [23] studied the impact of community health insurance on service utilisation and OOPs in Laos, and the results showed that people with health insurance used healthcare services significantly more than those without health insurance. Assessing the impact of community health insurance on access and quality of health services in Northern Ghana, [24] showed that the frequency of using services by insured people was nearly three times higher than that of uninsured people. Most people without health insurance (93.75 per cent) had to delay going to health facilities due to high costs, and they tried to get treatment at home and only went to health facilities when their illness was too serious to be treated at home. Similar findings were found by [25,26,27,28] for the effects of community health insurance programs on access to healthcare services in countries in Africa (such as Ghana, Rwanda, Senegal) and Asia (such as India).

However, the above benefits of SHI in Vietnam were not distributed equitably among older groups. This study showed that SHI could increase the use of outpatient services mainly for the younger and the richer groups than their other counterparts. This finding is similar to that from Ghana, where [29] found that about 67 per cent of the young-old (aged 60–69) and 100 per cent of those aged 70 years and older with health insurance used healthcare services at 10 times higher rates than the respective groups without health insurance. More notably, Kinh and urban older persons had significantly higher rates of using both outpatient and inpatient services than ethnic minority and rural older persons, respectively. These results are similar to those found by [14]. The gaps could be explained by the differences in the availability of healthcare services as well as perceived information about health and healthcare for older persons [30,31].

In regard to the financial burden of using healthcare services among insured and uninsured older persons, we found again that SHI helped Vietnamese older persons in reducing their financial burden. More specifically, the insured had 30.43 per cent and 41.22 per cent lower *OOPs* for outpatient services and inpatient services than the uninsured, respectively. This finding was similar to other studies for the general Vietnamese population, such as [32] which found that social health insurance helped the insured reduce their *OOPs* for healthcare services by about 16 to 18 per cent. Studies in other countries also provided similar findings. For instance, [33] assessed the impact of health insurance programs on OOPs in Indonesia and indicated that the two health insurance programs (i.e., Askekin and Askes) significantly reduced OOPs by 34 per cent and 55 per cent for people with health insurance, respectively. [34] assessed the impact of the World Bank’s Health Project VIII (including health insurance) in Gansu Province, China, using data in 2000 (the pre-project time) and in 2004 (the post-project time). They found that health insurance reduced both OOPs and the likelihood of falling into catastrophic spending, especially for the poorest quintile. Similar results were found by [26,29,35,36,37,38,39].

This research, however, also indicated that the benefits of reducing the financial burden of using healthcare services were not equally distributed between and within insured and uninsured older persons. Specifically, the oldest-old had lower *OOPs* for outpatient services but significantly higher *OOPs* for inpatient services than those for the middle-old and the young-old. Given the fact that the oldest-old have multiple morbidities and non-communicable diseases that require more inpatient admissions and long-term treatments, such a situation indicates that a heavier financial burden for the oldest-old is expected, and thus SHI should play a more critical role in covering the costs of healthcare for this vulnerable group of older persons. Although using different data sets, refs. [40,41] provided the same results as ours.

The SHI provided higher benefits for the ethnic minority groups—both the insured and uninsured—when using outpatient services than the Kinh group, but a contrasting situation was observed for inpatient services. Given the fact that inpatient services are usually more costly than outpatient services and that there is a lower quality of healthcare services in ethnic minority areas, such a situation implies that ethnic minority older persons—who are persistently disadvantaged in health status and accessing healthcare services [6,8,42]—did not really benefit from SHI. This was also presented by the finding of this research that, for both the insured and uninsured, households with ethnic minority older persons had higher rate of *CHEs* and thus a higher rate of impoverishment than the households with Kinh older persons. Although the rate of having SHI among ethnic minority older persons was higher than that of their Kinh counterparts (since the former group is fully subsidized in purchasing SHI cards), the aforementioned situation indicates that SHI could improve access to healthcare services but not financially protect ethnic minority older persons.

The finding in regard to the expenditure quintile in this research is also similar to that from [32], which showed that SHI could reduce OOPs by 16 per cent to 18.5 per cent, and the reduction was greater among the lower income earners.

With the rapid expansion of grassroot health facilities, particularly with more than 10,000 commune/ward health centres, it is not surprising to find no significant difference in the financial burden for urban and rural older persons—both the insured and uninsured—in outpatient services in this research. Indeed, the finding is similar to that of [43]. However, as inpatient services are more available at provincial and central level hospitals, the financial burden was heavier for rural persons than their urban counterparts. This was also found by [40,41]. For both the insured and uninsured, rural elderly households had higher rates of *CHEs* and thus higher rates of impoverishment than urban elderly households. Reducing *OOP*-related costs (such as transportation and accommodation) in utilising healthcare services for rural persons would help them reduce financial burden, particularly for inpatient admissions [41,43,44,45].

## 5. Conclusions

Using the nationally representative data from the Vietnam Household Living Standard (VHLSS) in 2014, this research compared insured and uninsured older persons under the SHI scheme in utilising outpatient and inpatient services as well as their financial burden in paying for these services. This research found that SHI could help insured older persons attain higher utilisation of both outpatient and inpatient services as well as lower their financial burden, particularly at catastrophic spending levels. However, it was critical that the benefits from SHI were not equitably distributed between and within the insured and uninsured. In particular, the more vulnerable groups, i.e., ethnic minorities and rural persons, had lower utilisation rates and higher rates of catastrophic spending for healthcare services than the better groups (i.e., Kinh and urban persons). This is an important message for health policymakers when designing a healthcare system along with the SHI mechanism to provide more equitable access and financial protection to all older persons in Vietnam. In order to reach such goals, we would suggest the following policies.

First, improve the quality of healthcare at the grassroots level and reduce the burden on the provincial and central health levels. It is necessary to improve the quality of healthcare at grassroots health facilities (i.e., district and commune levels) because these facilities are close to older people, and as such, time and related costs for healthcare access (such as food, accommodation, and transportation) can be reduced. Caregivers of older persons can also reduce OOP burdens and avoid decreased income due to interrupted work resulting from taking care of older patients who have treatments at higher-level healthcare facilities.

Second, improve human resources for the grassroots healthcare facilities. Having qualified and professional doctors at these facilities will increase the confidence of local people in general and older persons in particular in the quality of healthcare services at the grassroots level.

Third, encourage public–private partnerships (PPPs) in healthcare service provision. Experiences from pilot models in different provinces showed that PPPs would help increase access while providing affordable and adequate care services to older persons [30,31]. In addition to this, encouraging private healthcare facilities to provide aged care services paid for by social health insurance is also an important policy direction.

Fourth, develop a nationwide family doctor network. Since care for older persons is mostly provided at home, such a network is crucial for providing timely consultation services and initial treatments for older persons and their home-based caregivers. Particularly for older persons having chronic and non-communicable diseases, a network of family doctors will help them gain timely access and adequate healthcare services.

Although this research could provide various evidence-based implications for the government of Vietnam when revising the current Social Health Insurance Law, it could not avoid some limitations. Due to utilizing data from 2014, the results might not reflect all current changes in healthcare service provision paid for by health insurance, particularly those for older persons. As such, updating the results with recent data and exploring factors associated with out-of-pocket payments and catastrophic spending on healthcare for older persons should be conducted as the next steps of this research field.

## Figures and Tables

**Table 1:** Differences in utilization of healthcare services by the insured and uninsured, 2014.

	Outpatient Visits per Year				Inpatient Admissions per Year			
	The Insured	The Uninsured	Difference		The Insured	The Uninsured	Difference	
			Number of Visits	%			Number of Admissions	%
**Total**	3.65	2.90	0.75	25.86	0.93	0.27	0.66	244.44
**Age group**								
60–69	3.57	2.58	0.99	38.37	0.88	0.25	0.63	252.00
70–79	3.83	3.22	0.61	18.94	0.83	0.30	0.53	176.67
80+	3.57	3.49	0.08	2.29	1.16	0.29	0.87	300.00
**Ethnicity of household head**								
Ethnic minority	3.21	3.46	−0.25	−7.23	0.83	0.35	0.48	137.14
Kinh	4.40	2.87	1.53	53.31	0.94	0.26	0.68	261.54
**Place of residence**								
Rural	2.63	3.21	−0.58	22.05	0.91	0.28	0.63	225.00
Urban	3.49	4.40	−0.91	26.07	0.99	0.23	0.76	330.43
**Per capita expenditure quintile**								
Poorest	3.37	2.75	0.62	22.55	0.97	0.22	0.75	340.91
Near-poor	3.08	2.80	0.28	10.00	1.06	0.23	0.83	360.87
Middle	3.56	2.72	0.84	30.88	0.95	0.27	0.68	251.85
Near-rich	3.17	3.02	0.15	4.97	0.91	0.21	0.70	333.33
Richest	4.61	3.37	1.24	36.80	0.83	0.45	0.38	84.44
**Health facilities**								
Commune health centres	3.99	3.11	0.88	28.30	0.24	0.12	0.12	100.00
District hospitals	3.86	2.61	1.25	47.89	1.12	0.34	0.78	229.41
Provincial hospitals	3.13	2.42	0.71	29.34	1.22	0.46	0.76	165.52
Central hospitals	3.68	1.27	2.41	189.76	0.97	0.85	0.12	14.12
Private healthcare facilities	3.21	3.58	−0.37	−10.34	0.18	0.06	0.12	200.00
Other	3.11	3.71	−0.6	−16.17	0.53	0.04	0.16	1225.00

Source: Own calculations, using 2014 VHLSS data.

**Table 2:** Average out-of-pocket payments (OOPs) for healthcare services by the insured and uninsured, 2014.

	For Outpatient Visits				For Inpatient Admissions			
	The Insured (1000 VND)	The Uninsured (1000 VND)	Difference		The Insured (1000 VND)	The Uninsured (1000 VND)	Difference	
			(1000 VND)	(%)			(1000 VND)	%
**Total**	860.98	1.237.61	376.63	30.43	3877.28	6596.80	2.719.52	41.22
**Age group**								
60–69	808.92	1070.65	261.73	24.45	4393.78	5960.53	1566.75	26.29
70–79	1062.19	1245.89	183.70	14.74	3000.81	10.880.12	7.879.31	72.42
80+	683.32	1876.27	1192.95	63.58	4214.87	2935.43	−1.279.44	−43.59
**Ethnicity of household head**								
Ethnic minority	288.86	729.20	440.34	60.39	2.253.71	2.523.22	269.51	10.68
Kinh	911.68	1257.52	345.84	27.50	4018.00	6859.82	2.841.82	41.43
**Place of residence**								
Rural	727.62	1058.10	330.48	31.23	3.114.24	4748.73	1634.49	34.42
Urban	1075.33	1.616.44	541.11	33.48	5.549.12	13,047.93	7498.81	57.47
**Per capita expenditure quintile**								
Poorest	243.64	474.12	230.48	48.61	1.696.09	1606.54	−89.55	−5.57
Near-poor	600.38	725.03	124.65	17.19	2.023.40	3.249.04	1.225.64	37.72
Middle	952.95	1290.95	338	26.18	2014.83	3885.17	1870.34	48.14
Near-rich	792.59	1689.62	897.03	53.09	5373.31	9900.07	4526.76	45.72
Richest	1339.41	2394.60	1055.19	44.07	6.789.17	17,507.11	10,717.94	61.22
**Health facilities**								
Commune health centres	135.59	145.95	10.36	7.10	501.42	157.71	−343.71	−217.94
District hospitals	510.53	561.26	50.73	9.04	1679.02	2.172.04	493.02	22.70
Provincial hospitals	1.692.10	3097.58	1405.48	45.37	5.018.36	6443.62	1.425.26	22.12
Central hospitals	2.475.83	4592.93	2117.1	46.09	9308.58	18,989.42	9680.84	50.98
Private healthcare facilities	1069.31	944.82	−124.49	−13.18	7970.00	12,434.62	4.464.62	35.90
Other	1016.64	2.127.58	1.110.94	52.22	2860.13	757.31	−2102.82	−277.67

Source: Own calculations, using 2014 VHLSS data.

**Table 3:** Indicators for household health spending by the insured and uninsured, 2014.

	The Insured			The Uninsured		
	OOP/Person (1000 VND)	OOP/CTP (%)	OOP/EX (%)	OOP/Person (1000 VND)	OOP/CTP (%)	OOP/EX (%)
**Total**	2937.88	6.58	4.07	2200.82	5.08	3.08
**Ethnicity of household head**						
Ethnic minority	1314.80	5.96	3.07	1336.90	4.55	2.57
Kinh	3164.09	6.66	4.20	2236.45	5.11	3.10
**Place of residence**						
Rural	2744.58	7.57	4.50	2043.58	5.41	3.16
Urban	3302.16	4.70	3.24	2578.42	4.29	2.87
**Per capita expenditure quintile**						
Poorest	1157.86	6.73	3.42	808.58	4.17	2.10
Near-poor	1971.02	6.63	3.81	1603.71	5.67	3.08
Middle	2636.87	6.31	3.99	2163.84	5.54	3.33
Near-rich	4520.77	8.20	5.64	2691.39	4.87	3.30
Richest	5613.86	5.15	4.08	4837.64	4.84	3.78

Note: OOP—out-of-pocket payment; OOP/CTP: ratio of OOP to a household’s ability to pay (CTP); OOP/EX: ratio of OOP to total expenditure of elderly households. Source: Own calculations, using 2014 VHLSS data

**Table 4:** The ratio of OOP to a household’s ability to pay (CTP) by the insured and uninsured, 2014.

	The Insured				The Uninsured			
	OOP/CTP (%)				OOP/CTP (%)			
	>10%	>20%	>30%	>40%	>10%	>20%	>30%	>40%
**Total**	20.44	9.98	4.76	2.42	15.67	6.35	2.86	1.29
**Ethnicity of household head**								
Ethnic minority	17.40	9.29	5.61	2.24	9.63	9.63	1.68	0.01
Kinh	20.86	10.07	4.63	2.44	15.92	6.22	2.90	1.34
**Place of residence**								
Rural	23.31	12.29	5.93	2.95	17.15	6.72	2.84	1.50
Urban	15.02	5.62	2.52	1.40	12.10	5.46	2.90	0.78
**Per capita expenditure quintile**								
Poorest	22.41	9.20	5.25	3.04	13.70	5.39	1.64	0.73
Near-poor	21.74	10.84	4.00	1.33	18.28	7.27	3.06	1.29
Middle	19.46	10.64	2.69	2.12	16.58	6.60	3.00	2.17
Near-rich	24.06	14.03	7.83	2.73	14.95	5.63	2.61	0.89
Richest	13.90	6.41	3.96	2.50	13.06	6.72	4.44	1.12

Source: Own calculations, using 2014 VHLSS data.

**Table 5:** Percentage of elderly households suffering from poverty due to health expenditure by health insurance status, 2014 (%).

	The Insured	The Uninsured
**Total**	1.77	1.04
**Ethnicity of household head**		
Ethnic minority	2.90	3.78
Kinh	1.62	0.92
**Place of residence**		
Rural	2.45	1.47
Urban	0.50	0.00

Source: Own calculations, using 2014 VHLSS data.

## Data Availability

Source data are available through the General Statistics Office of Vietnam (GSO) website. All data are presented in the manuscript.
